# Prediction of treatment outcome in neovascular age-related macular degeneration using a novel convolutional neural network

**DOI:** 10.1038/s41598-022-09642-7

**Published:** 2022-04-07

**Authors:** Tsai-Chu Yeh, An-Chun Luo, Yu-Shan Deng, Yu-Hsien Lee, Shih-Jen Chen, Po-Han Chang, Chun-Ju Lin, Ming-Chi Tai, Yu-Bai Chou

**Affiliations:** 1 Department of Ophthalmology, Taipei Veterans General Hospital, No. 201, Sec.2, Shih-Pai Road, Taipei, 11217 Taiwan; 2 National Yang Ming Chiao Tung University, Taipei, Taiwan; 3 Industrial Technology Research Institute, Hsinchu, Taiwan; 4 National Tsing-Hua University, Taipei, Taiwan

**Keywords:** Diseases, Medical research

## Abstract

While prognosis and risk of progression are crucial in developing precise therapeutic strategy in neovascular age-related macular degeneration (nAMD), limited predictive tools are available. We proposed a novel deep convolutional neural network that enables feature extraction through image and non-image data integration to seize imperative information and achieve highly accurate outcome prediction. The Heterogeneous Data Fusion Net (HDF-Net) was designed to predict visual acuity (VA) outcome (improvement ≥ 2 line or not) at 12th months after anti-VEGF treatment. A set of pre-treatment optical coherence tomography (OCT) image and non-image demographic features were employed as input data and the corresponding 12th-month post-treatment VA as the target data to train, validate, and test the HDF-Net. This newly designed HDF-Net demonstrated an AUC of 0.989 (95% CI 0.970–0.999), accuracy of 0.936 [95% confidence interval (CI) 0.889–0.964], sensitivity of 0.933 (95% CI 0.841–0.974), and specificity of 0.938 (95% CI 0.877–0.969). By simulating the clinical decision process with mixed pre-treatment information from raw OCT images and numeric data, HDF-Net demonstrated promising performance in predicting individualized treatment outcome. The results highlight the potential of deep learning to simultaneously process a broad range of clinical data to weigh and leverage the complete information of the patient. This novel approach is an important step toward real-world personalized therapeutic strategy for typical nAMD.

## Introduction

Age-related macular degeneration (AMD) is a leading cause of irreversible blindness worldwide, and the number of affected population is expected to reach 288 million by 2040^1^. It is a debilitating, chronic and progressive disease that independence and overall quality of life decline in parallel with visual impairment^2^. Over the past several years, anti-vascular endothelial growth factor (anti-VEGF) injection has become the mainstay of treatment for neovascular AMD (nAMD), and has significantly improved visual outcomes and prevent vision loss in most patients^3^. However, high cost and the need for frequent injections result in substantial healthcare burden on both patients and physicians^4,5^. In addition, questions persist regarding whether an intensive anti-VEGF therapy may have negative effects on nonvascular tissues and influence in the retinal pigment epithelium (RPE) and choriocapillaris integrity^6–8^. Moreover, each injection poses a small yet significant risk of complications threatening eyesight including endophthalmitis, retinal tear or detachment, and vitreous hemorrhage^9^. All these staggering disease burden and potential risks have prompted search for a more precise approach for dosage minimization.

Artificial intelligence (AI) has played an increasingly prominent role in every aspect of ophthalmology in recent years^10,11^. With the great potential of imitating the neural structure of the brain, deep learning has risen to the forefront in healthcare. Inspired by the structure of the primary visual cortex, convolutional neural network (CNN) represents the latest revolution of deep learning technologies, which is capable of effectively analyzing medical images^12^. Besides automated detection of features, successful application of AI has been reported for individualized treatment requirements and outcome prediction. Bogunovic et al. evaluated a total of 317 subjects with OCT images from baseline, month 1, and month 2 to predict anti-VEGF injection requirements^13^. Schmidt-Erfurth et al. introduced a prognostic model using random forest machine learning to predict visual outcomes after 12 months of anti-VEGF injection in the setting of a randomized controlled trial^14^. Rohm et al. proposed a model for visual acuity (VA) prediction based on clinical data from electronic medical records (EMR) and measurement features from OCT^15^. Although prior studies have presented proof-of-principle evidence, longitudinal datasets and serial OCT images are often required to make predictions. Furthermore, previous deep learning models were often developed based on pre-defined extracted features associated with nAMD that were confirmed to be clinically relevant in literatures. CNN has been proven very effective for image classifications, thus, medical image analysis is one of the early applications of CNNs in healthcare. Nonetheless, even for imaging-based medical specialties, clinical data is crucial to guide image interpretation and clinical practice. Therefore, multimodal deep learning models that can ingest both image and clinical data have shown an increased role in healthcare.

The aim of this study is to introduce a novel CNN architecture automatically and simultaneously process real-world image and non-image data for VA outcome prediction after 12-months of anti-VEGF treatment. Since it is of great interest to be able to predict treatment outcomes for each patient at the very beginning of the therapeutic course, we use only basic patient demographics, baseline OCT image and baseline BCVA. An important additional aim of this study is to compare the accuracy with traditional CNN algorithm to unleash the potential of deep learning and facilitate resource management, therapeutic decision making, and patient counseling.

## Materials and methods

### Data sources and study population

We retrospectively reviewed the medical records of patients with nAMD who underwent an intravitreal injection (IVI) of anti-VEGF drugs in the interval of three consecutive monthly injections and pro re nata injections (PRN) at the Taipei Veterans General Hospital from November 2013 to July 2018. The inclusion criteria were as follows: (1) age ≥ 55 years; (2) a diagnosis of typical nAMD confirmed with fluorescein angiography (FA), indocyanine green angiography (ICGA), or OCT angiography; (3) no documented IVI of anti-VEGF within 6 months prior to study entry; (4) ﻿Patients with one-year follow-up data available. The exclusion criteria were: (1) Patients with other intraocular vascular, inflammatory, infective, or ischemic diseases such as polypoidal choroidal vasculopathy, uveitis, or retinal vein occlusion…etc.; (2) Patients with history of intraocular operation other than IVI within the following 12-month treatment period, including cataract surgery and vitreoretinal surgery. This study followed the tenets of the Declaration of Helsinki and was approved by the Institutional Review Board of the Taipei Veterans General Hospital.

### Data and OCT image pre-processing

The pretherapeutic spectral-domain (SD) OCT images were acquired at baseline with AngioVue Imaging System (RTVue-SD OCT; Optovue Inc, Fremont, CA, USA). The OCT images consisted of the 10-mm horizontal and vertical B-scans of the cross line report of the macular scan, which were cut and resized to 620 × 620 pixel centered at fovea using the region of interest (ROI) cutting technique. The two images of the same eye were allocated into the same dataset.

**Figure 1:**
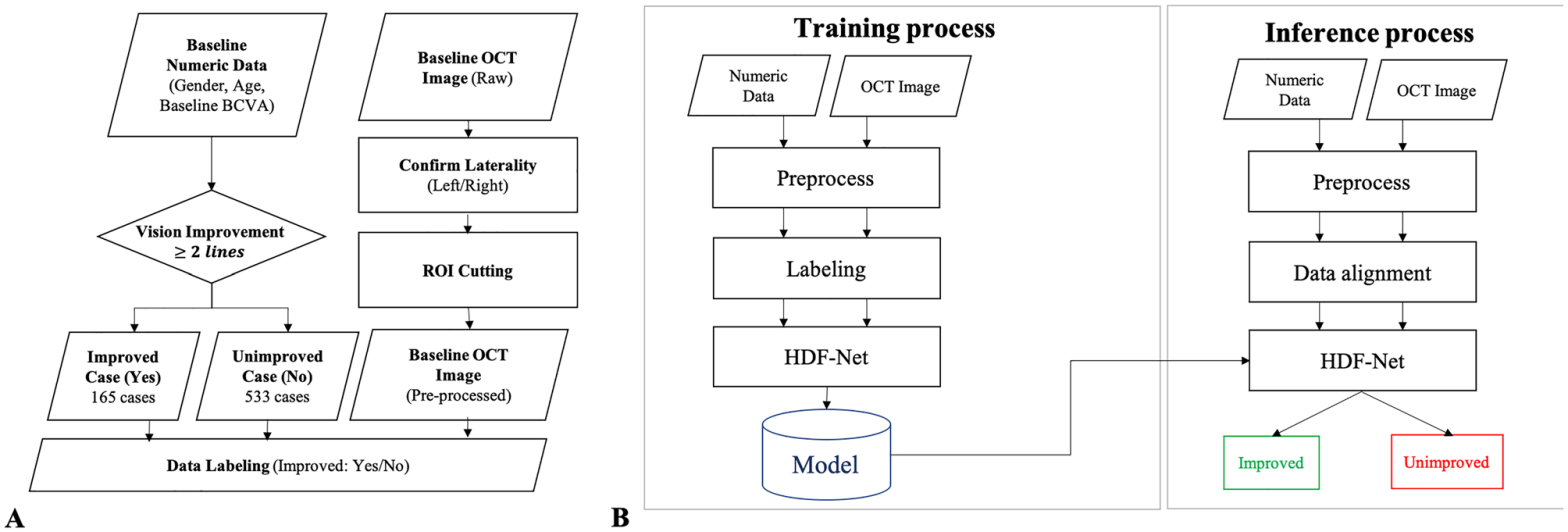
(**A**) Illustration of the pre-process flow. (**B**) The flowchart of the HDF-Net process.

For patient demographics and clinical data, baseline best-corrected visual acuity (BCVA), gender, and age were extracted from the electronic medical records. SD-OCT scans were preprocessed using motion artifact removal to reduce image artifacts caused involuntary eye motion during acquisition. OCT images were randomly separated into the training, validation and testing dataset. There were no images of the same patient simultaneously assigned to the two datasets. The analysis of visual outcome is evaluated at baseline and 12th months after treatment. We defined the “improved case” to consist of patients with visual acuity increase ≥ 2 lines of Snellen chart at 12th month after treatment. Patients with visual acuity increase < 2 lines were defined as “unimproved case” (Fig. 1A).

### Overview of the AI model

HDF-Net was designed as a deep learning model that could predict treatment outcome using baseline clinical data and OCT image by Industrial Technology Research Institute of Taiwan (Fig. 1B). The HDF-Net described herein was granted provisional patent 63/091,280 on Oct. 13th, 2020.

**Figure 2:**
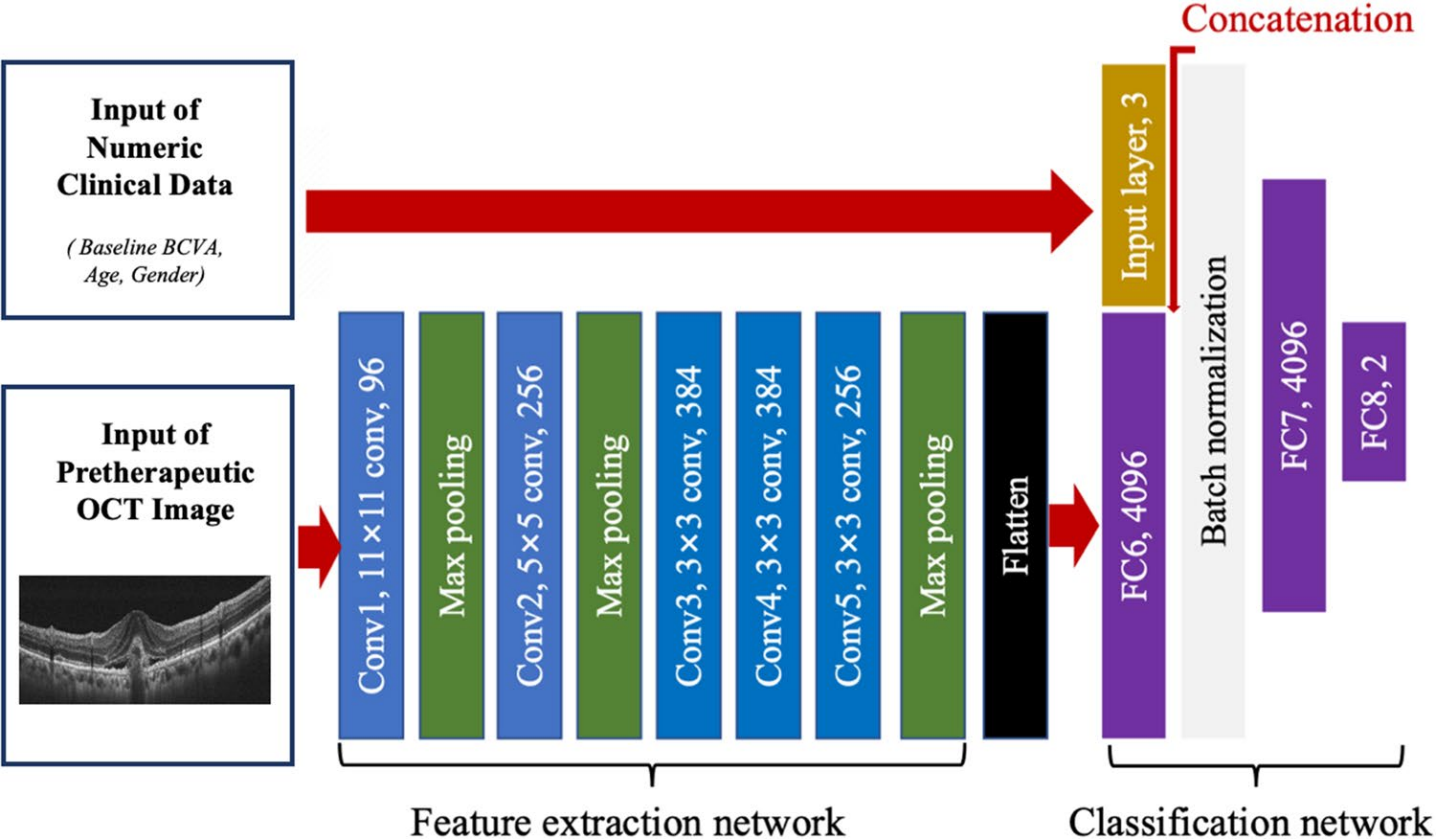
The architecture of HDF-Net. The feature extraction network consists of five convolution layers. The first convolutional layer has 96 kernels within the size of 11 × 11, presented as "Conv1, 11 × 11 conv, 96", while the second convolutional layer has 256 kernels within the size of 5 × 5, the third and fourth convolutional layer has 384 kernels within the size of 3 × 3, and the fifth convolutional layer has 256 kernels within the size of 3 × 3.

Contrary to conventional classification approach in which extracted features are fed into algorithms, HDF-Net automatically learn representative complex features directly from the image and numeric data itself. The overall structure of the HDF-Net is shown in Fig. 2. The baseline OCT image and clinical data are the input dataset to the HDF-Net. The first stage of HDF-Net is the image feature extraction network, which consists of five convolutional layers (from Conv1 to Conv5) and three maximum pooling layers. Output signal of the final pooling layer, which is the feature map extracted from the OCT image, is flatten to be reshaped into a vector as the input signal of the next stage. The classification network consists of two fully connected layers (FC6 and FC7) with a dropout probability of 0.5 and a final 1 × 2 softmax layer (FC8, output layer) served as a two-class classifier. An addition input layer is designed for the corresponding numeric data, and is concatenated with the layer FC6. Layer FC7 hybridizes the image features extracted from the first stage and the numeric features from baseline patient data. Rectified linear unit (ReLU), the most commonly used activation function, is applied after each hidden layer. To normalize layer inputs, a batch normalization (BN) layer is added after layer FC6.

### Performance comparison between HDF-Net, ResNet50 and AlexNet

﻿The performance of the three models (HDF-Net, ResNet50^16^ and AlexNet^17^) was evaluated using cross-validation techniques with the dataset split randomly into 80% and 20% for training and testing sets, respectively. The AlexNet classifier was trained with baseline OCT image dataset using Caffe deep learning framework. The transfer learning technique was applied by initializing the five convolutional layers of AlexNet with the weights pre-trained on ImageNet, and the base learning rate was set to 0.001 for the stochastic gradient descent (SGD) to re-train the whole AlexNet model with a batch size of 100. In comparison, we trained HDF-Net with heterogeneous dataset including not only baseline OCT images but the corresponding numeric clinical data (pretherapeutic BCVA, gender, and age). The batch size and the base learning rate were the same as those set in the training process of ResNet50 and AlexNet.

### Evaluation of model and statistical analysis

To evaluate the performance and to assess how the results would generalize to an independent data set, we used a holdout cross validation method. We randomly assign data points to the training and testing dataset, following a training/validation splitting ratio of 0.75/0.25. The quantitative performance of the two predictive models across all the validated predictions is summarized with an area under the receiver operating characteristic (ROC) curve and presented as sensitivity and specificity at an operating point.

### Ethical statement

The authors are accountable for all aspects of the work in ensuring that questions related to the accuracy or integrity of any part of the work are appropriately investigated and resolved.

### Informed consent statement

Patient consent was waived due to the privacy rule, as deemed by the Institutional Review Board.

## Results

### Patient characteristics

The demographics of all patients/eyes included are summarized in Table 1. Of all 698 evaluable patients, 232 were male (33.24%) and 466 were female (66.76%). The mean (± SD) age of patients was 78.47 (± 9.88) years. 165 patients had at least 2-line VA improvement, and 533 failed to achieve VA improvement ≥ 2 line. The mean baseline BCVA of the “improved cases” and “unimproved cases” was 0.32 ± 0.15 and 0.19 ± 0.15, respectively; The mean 12th-month BCVA was 0.62 ± 0.16 and 0.16 ± 0.16, respectively, after treatment.

**Table 1:** Patient demographics.

	Total	Improved Group (VA increase ≥ 2 lines)	Unimproved Group (VA increase < 2 lines)
**Case number**	698	165	533
Treatment-naïve (%)	467 (66.91%)	103 (62.42%)	364 (68.29%)
Non-treatment-naïve (%)	231 (33.09%)	62 (37.58%)	169 (31.71%)
Age years (± SD)	78.47** ± **9.88	78.47** ± **9.88	83.28** ± **9.94
**Gender**			
Male (%)	466 (66.76%)	97 (58.79%)	369(69.23%)
Female (%)	232 (33.24%)	68 (41.21%)	164(30.77%)
Pre-therapeutic BCVA (mean ± SD)	0.21** ± **0.16	0.32** ± **0.15	0.19** ± **0.15
12th month Post-therapeutic BCVA (mean ± SD)	0.19** ± **0.19	0.62** ± **0.16	0.16** ± **0.16
Anti-VEGF injections in 12 months (mean ± SD)	4.19 ± 2.11	4.50 ± 2.15	4.18 ± 2.11
**Anti-VEGF agent**			
Aflibercept (%)	544 (77.94%)	134 (81.21%)	410 (76.92%)
Ranibizumab (%)	154 (22.06%)	31 (18.79%)	123 (23.08%)

### Prediction of visual outcomes

**Figure 3:**
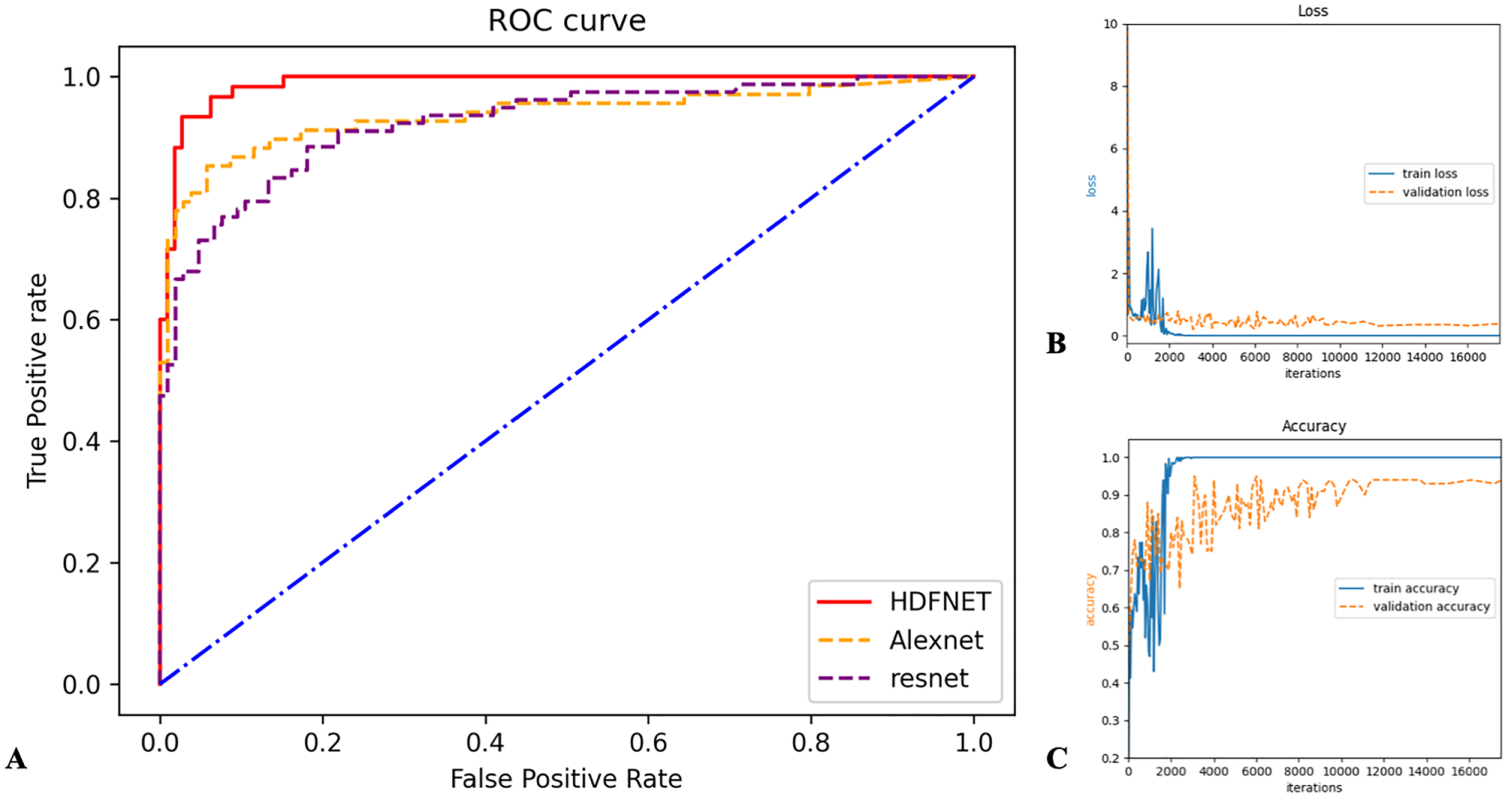
Receiver operating characteristic (ROC) curves and learning curve of the HDF-Net, ResNet50 and AlexNet for VA outcome prediction. (**A**) For binary classification tasks of VA improved or unimproved, the presented HDF-Net (red line) performs equally or better than the traditional AlexNet (orange line) and ResNet50 (purple line). (**B**) Loss versus iteration graph of HDF-Net showing loss of 0.351 at 16,000 iteration; (**C**) Accuracy versus iteration graph of HDF-Net showing a final validation accuracy of 93.6% at 16,000 iteration.

We evaluated the performance of the model in an internal validation set. ﻿A total of 1396 image series representing 698 individuals was successfully exported from the image archive. 75% were used for model training. The HDF-Net predicts the one-year VA outcome with AUC of 0.989 (95% CI 0.970–0.999), accuracy of 0.936 (95% confidence interval [CI] 0.889–0.964), sensitivity of 0.933 (95% CI 0.841–0.974), and specificity of 0.938 (95% CI 0.877–0.969). While the metrics for ResNet50 were AUC of 0.924 (95% CI 0.870–0.934), accuracy of 0.852 (95% CI 0.841–0.933) sensitivity of 0.795 (95% CI 0.716–0.896), and specificity of 0.895 (95% CI 0.822–0.952); and the metrics for AlexNet were AUC of 0.936 (95% CI 0.894–0.978), accuracy of 0.895 (95% CI 0.841–0.933), sensitivity of 0.824 (95% CI 0.716–0.896), and specificity of 0.942 (95% CI 0.880–0.973). Figure 3 describes the area under the receiver operating characteristic (ROC) curve (AUC) and demonstrates learning curves of internal validation.

### Attention maps generated on OCT images by HDF-Net

**Figure 4:**
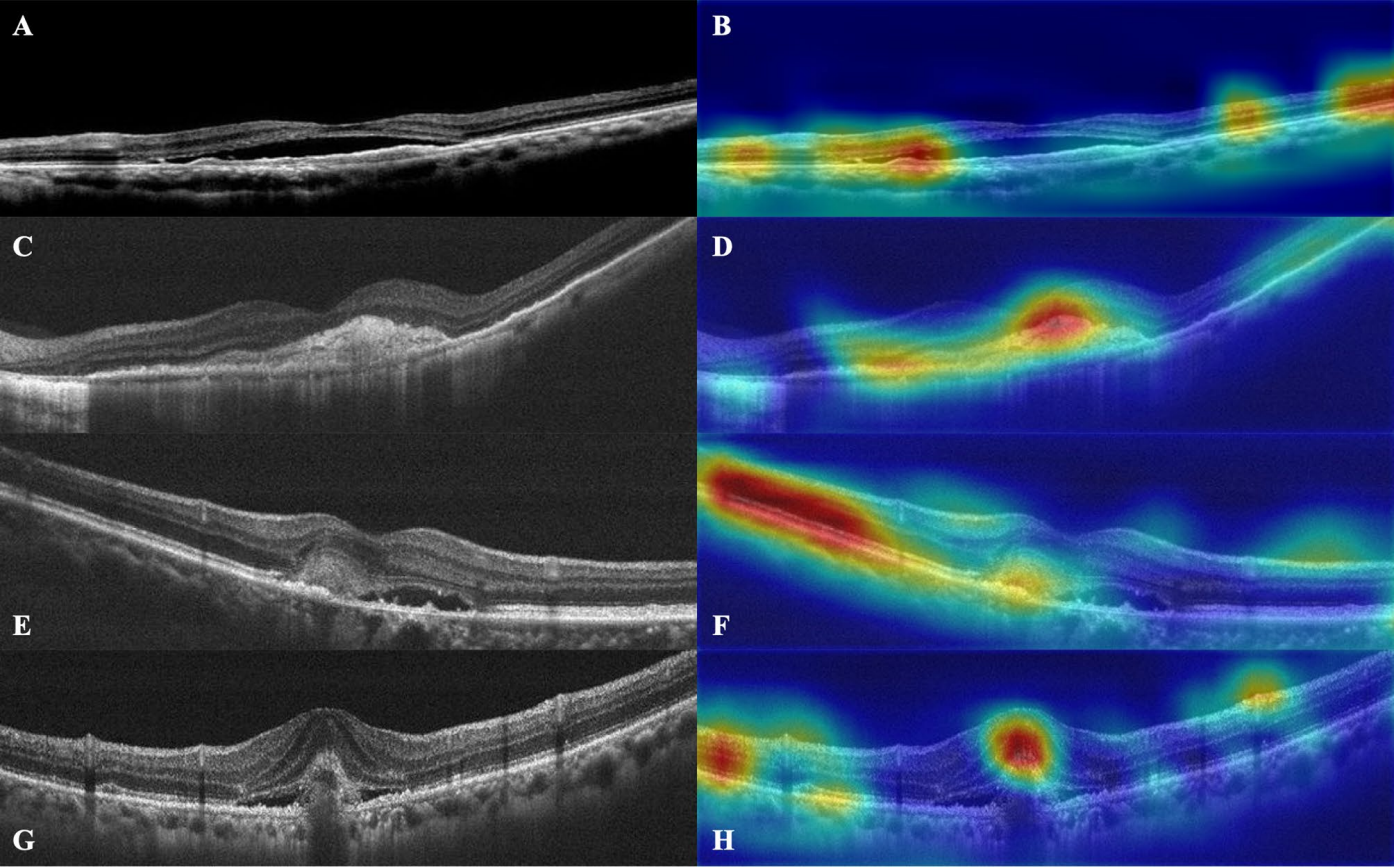
Representative horizontal scans of SD-OCT and corresponding superimposed heatmaps. Presented are (**A**) an example of an OCT image with the HDF-Net correctly predicted as an improved case (85 M; baseline VA:0.2; 12th month VA: 0.5); (**B**) The network located attention on the margin of SRF, and on retinal pigment epithelial mottling. Also, some attention corresponded to islands of preserved ellipsoid zone (EZ) reflecting preserved visual potential. (**C**) An example of an OCT image with the HDF-Net correctly predicted as an unimproved case (94 M; baseline VA:0.05; 12th month VA: 0.1); (**D**) The attention is located at the subfoveal disciform scar and disruption of the EZ. There is also some attention on the chorioretinal atrophy with adjacent loss of outer retinal layers. (**E**) An example of an OCT image with the HDF-Net predicted to improve but the patient failed to achieve VA improvement >  = 2 lines (96 M; baseline VA:0.4; 12th month VA: 0.4); (**F**) The attention is located primarily on the preserved EZ and the subretinal hyperreflective material (SHRM) with fibrovascular components. A relatively normal fovea contour was also identified. (**G**) An example of an OCT image with the HDF-Net predicted not to improve but the patient demonstrated VA improvement >  = 2 lines (82 M; baseline VA:0.4; 12th month VA: 0.6); (**H**) The attention is located on the SHRM with fibrovascular/hemorrhagic components. Also some attention on the disruption of the EZ and the hyperreflective zone of the inner retina.

Consequent to the evaluation of OCT images using the HDF-Net, attention maps were generated and overlaid on the OCT images to represent in a quantitative manner the relative contributions that areas in each image made to the ascertainment decision. Moving on to model explanation, heatmaps demonstrated that areas contributed most consequentially to visual outcome identified by HDF-Net are fovea contour, the ellipsoid zone (EZ), and other pathological features associated with nAMD, in agreement with what clinicians deem relevant in prognosis. Examples of these attention maps representing true and false outcome forecast are shown in Fig. 4. This verification allows us to safely suggest that the HDF-Net displays a degree of validity and is making classifications based on anatomical integrity and pathological features rather than systemic errors that cannot be explained. A common classification error is seen on OCT images with subretinal hyperreflective material (SHRM), which may be a collection of neovascular tissue, fibrosis, exudate or hemorrhage.

## Discussion

AI is making precision medicine a reality, strengthened by digital healthcare revolution including integrated electronic health records and advancement of computational power. In fact, prediction is not new to ophthalmology. A variety of risk scores have been investigated to determine individual risk for different ocular diseases, in search for a personalized medicine approach. However, it was only until the rise of deep learning algorithms that the concept of forecasting precise treatment outcome can be realized. Our study demonstrates that a deep learning neural network was effective at predicting one year treatment outcome from baseline OCT images (AlexNet accuracy: 0.895, sensitivity: 0.824, specificity: 0.942, AUC: 0.936), and the accuracy was even higher when clinical data were combined using a novel CNN model (HDF-Net accuracy: 0.936, sensitivity: 0.933, specificity: 0.938, AUC: 0.989).

The prediction of nAMD visual outcome derived from OCT features has been previously studied by other groups^13,18^. For instance, Schmidt-Erfurth et al. introduced a model to predict visual outcomes in the setting of a randomized controlled trial, and demonstrated R^2^ = 0.34 if only the baseline data was considered^14^; Rohm and colleagues developed and validated a model in 456 patients, and showed successful VA prediction within an error margin of 8 letters after one year of real-world anti-VEGF therapy^15^. All of these studies were particularly comprehensive in including “pre-defined” OCT measurement data, such as central retinal thickness, subretinal fluid (SRF) and intraretinal fluid (IRF), into the machine learning model. However, some important anatomic aspects had been missed out since they were difficult to capture in currently available automated segmentation methods. In fact, the conventional machine learning approach such as random forest requires the input data to be in the form of a feature vector instead of an OCT image itself, which makes the whole process highly time-consuming in feature labelling and highly dependent on the feature extraction technique^19^.

Based on prior evidence, baseline BCVA has become an important prognostic factor for final visual outcomes^20^. However, to date, automated analysis of “raw” retinal OCT images in combination with baseline BCVA in a single CNN model has not yet been explored to predict treatment outcome in nAMD. To address these limitations, we developed the HDF-Net to predict visual outcome simply by using baseline OCT image and three demographic covariates (age, gender, and baseline BCVA). In contrast to traditional machine learning approach, CNN model accepts a sample as an image and performs feature extraction and classification via hidden layers. But the challenges remain in how to input hybrid heterogeneous image and non-image data into a single CNN architecture. Unlike conventional CNN approaches which can only allow single type of data as input, we used a data fusion approach that enables simultaneous processing of multiple types of data with heterogeneous features extracted from different sources. Thereby, the power of CNN can be unleashed for image and non-image data all at the same time.

The difference between HDF-net and other published data fusion CNN approaches, such as HDF-CNN is the input method of numeric data. The HDF-CNN uses the matrix format to perform heterogeneous data fusion^21^. Hence, all heterogeneous data becomes a single input instance, and the heterogeneous data features are extracted through CNN. However, there may be some information loss in numeric data after the convolutions and pooling. Furthermore, pertinent features among numeric data extracted by convolution layers may turn out to be insignificant. In contrast, the HDF-net we proposed here is designed to merge image features and numeric data into a single feature vector after the feature extraction layers. The classification layer of HDF-Net will later on automatically determine the feature weight among various input data. In this work, we showed that the HDF-Net approach is superior to models such as ResNet50 and AlexNet at accurately predicting VA outcome in a real-life population. HDF-Net encompasses many advantages including automatic feature extraction in non-labeled samples, finding hidden structure from sparse and hyper-dimensional data, and non-image data hybridization. Therefore, it holds the potential to offer a robust decision support with non-image data integration as genetic factors are known to be involved in determining nAMD prognosis^22,23^.

A strength of this study is that it is a model validated based on real-world population comprising not only treatment-naive individuals. Previous studies regarding AI predicting treatment outcome frequently used trial dataset, such as that from the HARBOR study, as it offers a standardized imaging data and well-designed treatment protocol from a large sample size. Nonetheless, real-world nAMD studies showed discrepancies in several aspects when compared to randomized controlled trials (RCTs). An analysis of 49,485 eyes assessing anti-VEGF intensity and VA change found that real-world nAMD patients receive fewer injections and experience worse visual outcomes compared with patients receiving fixed, frequent therapy in RCTs. Furthermore, patients with older age and poor baseline VA may be particularly prone to undertreatment^24^. This suggests potential bias in the results of studies validating AI algorithms only in trial settings. Although this study was designed as retrospective, all included patients were approved for Taiwan National Health Insurance (NHI) reimbursed anti-VEGF injections after a scrutinized cross-check on clinical diagnosis and OCT images from the Bureau of NHI. This further supports the diagnosis accuracy and standardized treatment protocol following the reimbursement scheme.

Another major strength of this study is that it aims far beyond identifying “pre-defined OCT features” but generates prediction rules from “raw OCT images”. In a study comparing the performance of retinal specialists and an AI algorithm, retinal specialists were found to have imperfect accuracy and low sensitivity in detecting retinal fluid whereas AI achieved a higher level of accuracy^25^. This supports our hypothesis that feeding AI with only “pre-defined OCT features” could limit the scope of its application as it became obvious that AI might be able to outperform human intelligence. Prior studies have demonstrated favorable accuracy of deep learning approach to detect “pre-defined OCT features” such as retinal fluid on OCT scans^25,26^, yet quantifying the retinal fluid cannot directly guide clinical practice as controversies remain in SRF tolerance after initiation of treatment^27–29^. Treating nAMD with an ultimate goal of completely drying the retina could also increase the risk of macular atrophy, causing deteriorated long-term visual outcomes^8,30^.

This study has some limitations and there are several factors to be improved to optimize the results. A major limitation of most deep-learning models is the issue of “black-box” indicating that their predictions might be hard to interpret. In this study, we applied heatmaps to localize image regions influencing the classification. Although heatmap is a useful clue to highlight which part of image guided the CNN model to its decision, it does not provide information about the reason behind it. By processing both image and non-image data in one single CNN architecture, we found that adding numeric clinical data from baseline might enhance the performance of the model. But, the optimal feature weighting among image and non-image data remains as a question of major interest. Another limitation is the relatively small sample size that may have jeopardized our statistical analysis. However, each participant was comprehensively assessed with horizontal and vertical OCT scans, providing a rich set of reliable data.

The therapeutic response of nAMD varies widely in real-world setting, and was difficult to predict in the past. In this study, we presented and validated a novel deep learning-based approach utilizing baseline OCT and clinical information including baseline BCVA to predict the 12-month visual outcome after standard anti-VEGF therapy for active nAMD. The combination of heterogenous clinical and image data in HDF-Net holds the potential to serves as a solid decision support tool for clinicians to deliver evidence-based personalized treatment. This breakthrough marks a new era in AI guiding treatment decisions and patient expectations. Future studies are warranted to evaluate both economic impact and patient perceptions regarding outcome predictions.

## Data Availability

The data presented in this study are available on request from the corresponding author. The data are not publicly available due to patient privacy IRB requirement.
